# Adolescent overeating and binge eating behavior in relation to subsequent cardiometabolic risk outcomes: a prospective cohort study

**DOI:** 10.1186/s40337-022-00660-4

**Published:** 2022-09-13

**Authors:** Joyce C. Zhou, Sheryl L. Rifas-Shiman, Jess Haines, Kathryn Jones, Emily Oken

**Affiliations:** 1 Harvard Medical School, 25 Shattuck St, Boston, MA 02115 USA; 2 Present Address: Department of Medicine, Massachusetts General Hospital, 55 Fruit Street, Boston, MA 02114 USA; 3 Division of Chronic Disease Research Across the Lifecourse, Department of Population Medicine, Harvard Medical School and Harvard Pilgrim Health Care Institute, 401 Park Drive, Suite 401 East, Boston, MA 02215 USA; 4 Department of Family Relations and Applied Nutrition, University of Guelph, 50 Stone Road East, Guelph, ON N1G 2W1 Canada; 5 Department of Nutrition, Harvard T. H. Chan School of Public Health, Boston, MA USA

**Keywords:** Binge eating behavior, Overeating behavior, Adolescence, Adiposity, Inflammation, Adipokines, Cardiovascular risk

## Abstract

**Background:**

Binge eating disorder is bidirectionally associated with obesity and with metabolic syndrome. It is less clear whether overeating and binge eating, or overeating with loss of control, also predicts metabolic risk, and if so, whether these associations are solely attributable to greater weight. The goal of this study was to examine longitudinal associations of overeating and binge eating behavior with cardiometabolic risk markers in adolescence.

**Methods:**

Adolescents (*n* = 619) in the Project Viva research study self-reported overeating and binge eating behavior in early adolescence (median 12.9 years, “baseline”). In late adolescence (median 17.4 years, “follow-up”), we assessed outcomes of adiposity and blood pressure, and in a subset of participants (*n* = 270–424), biomarkers of dyslipidemia, insulin resistance, liver dysfunction, inflammation, and adipokine homeostasis. We conducted multivariable linear regression analyses adjusted for socio-demographics and prenatal obesogenic exposures, and additionally for baseline body mass index (BMI) z-score.

**Results:**

At baseline, 58 (9%) participants reported overeating behavior, and of those, 24 (41%) had binge eating behavior (e.g., overeating accompanied by loss of control). In adjusted models, adolescents with overeating had higher adiposity at follow-up ~ 5 years later (e.g., % body fat 4.03; 95% confidence interval (CI) 1.76, 6.31) than those not reporting overeating behavior; additional adjustment for baseline BMI z-score attenuated associations generally except for % body fat (2.95; 95% CI 1.03, 4.87). Overeating behavior was also associated with higher inflammation and greater adipokine dysfunction, remaining positively associated with interleukin-6 (IL-6) (log-transformed β = 0.42 pg/mL; 95% CI 0.12, 0.73) and negatively with adiponectin (log-transformed β = −0.28 ug/mL; 95% CI − 0.47, − 0.08) even after adjusting for baseline BMI z-score. Overeating behavior was not consistently associated with other outcomes. Adolescents reporting binge eating behavior generally had the greatest adiposity, (e.g., % body fat 5.00; 95% CI 1.74, 8.25) as compared to those without overeating.

**Conclusions:**

Adolescents reporting overeating and binge eating behavior had higher adiposity and poorer inflammatory and adipokine profiles, but no difference in other outcomes, than adolescents who did not endorse these behaviors. These associations were only partially accounted for by higher baseline BMI z-score. These differences may signal increased risk for future cardiovascular disease.

**Supplementary Information:**

The online version contains supplementary material available at 10.1186/s40337-022-00660-4.

## Plain English summary

We examined associations of overeating and binge eating behavior risk markers for future heart disease and diabetes. Adolescents (*n* = 619) in the Project Viva research study self-reported overeating and binge eating behavior on questionnaires completed in early adolescence (~ 13 years, “baseline”). In late adolescence (~ 17 years, “follow-up”), we collected research measures of body fat and blood pressure, and in a subset of participants, blood levels of cholesterol, fat-related hormones, liver dysfunction, and inflammation. We applied analytic methods to adjust for socio-demographics and to better understand how baseline weight could explain the associations. At baseline, 58 (9%) participants reported overeating behavior, and of those, 24 (41%) had binge eating behavior (e.g., overeating behavior accompanied by feeling loss of control). We found that adolescents reporting overeating behavior had higher later body fat and poorer inflammatory and fat hormone concentrations than those who did not report overeating. These associations were only partially explained by the fact that those with overeating also had higher baseline weight. Other markers of cardiometabolic risk in late adolescence were not different among those with or without overeating. Overall, our study suggests that overeating and binge eating behavior are associated with some higher markers of heart disease and diabetes risk.

**Supplementary Information:**

The online version contains supplementary material available at 10.1186/s40337-022-00660-4.

## Background

Binge eating disorder, defined by recurrent, psychologically distressing episodes of consuming large amounts of food with loss of control (LOC) [1], is the most common eating disorder, with a lifetime prevalence of 3% [2]. Binge eating disorder is strongly associated with obesity bidirectionally and has been associated with metabolic syndrome [3, 4], a constellation of conditions that raise cardiovascular disease risk including impaired glucose tolerance, dyslipidemia, and hypertension. Binge eating disorder may also be associated with poorer inflammatory and adipokine profiles [5], which independent of metabolic syndrome, are likely atherogenic [6, 7]. Studies have inconsistently demonstrated whether the association between binge eating disorder and higher cardiometabolic risk is solely attributable to greater weight [3, 4].

Disordered eating behaviors such as overeating (eating an unusually large amount of food) and binge eating (overeating with LOC), often emerge in adolescence, a period of psychological and physiologic change over a background of increasingly prevalent dieting culture and pressure towards thinness [8, 9]. Like binge eating disorder, these disordered eating behaviors have been correlated with greater adiposity [10–13]. Fewer studies have examined the association between these eating behaviors and cardiometabolic risk markers, however. While overeating is inclusive of and more prevalent than binge eating behavior, rare studies examine whether adolescent overeating behaviors can impact cardiometabolic health trajectories [13]. Prior research has primarily focused on binge eating behavior: one cross-sectional study found that adolescents reporting binge eating behavior had higher blood pressure and low-density lipoprotein (LDL) cholesterol than those who did not, independent of body mass index (BMI), but no difference in composite metabolic syndrome rates [14]. Another longitudinal study found that children endorsing binge eating behavior had greater odds of developing metabolic syndrome five years later, independent of interim weight change [11]. While these studies lay a foundation for understanding the relationship between binge eating behavior and cardiometabolic consequences, both included study samples that selected for individuals with overweight and obesity and reported inconsistent associations with specific metabolic risk factors. The degree to which these associations are independent of higher baseline adiposity also remains unclear [3, 4, 10].

Thus, our study sought to replicate and expand on previous findings utilizing data from Project Viva, a large prospective cohort study in the United States. This study included adolescents with and without overeating and binge eating behaviors without specifically enrolling adolescents at risk for overweight or obesity. Additionally, we integrated parental data and baseline BMI, allowing for deeper consideration of biologic and shared environmental confounders. We hypothesized that early adolescent overeating and binge eating behavior would be associated with poorer late adolescent cardiometabolic risk markers after controlling for confounders, and that overeating and binge eating behavior conferred metabolic risk beyond that attributable to differences in BMI alone. We hypothesized that associations would be in a similar direction, but stronger for binge eating behavior compared with overeating. We were also interested in exploring whether there might be mediation of the observed relationship between binge eating behavior and cardiometabolic outcomes by body mass index over follow-up, and whether associations might differ by sex, pubertal stage, or baseline weight class.

## Methods

Project Viva is a prospective, pre-birth longitudinal cohort study recruited from Atrius Harvard Vanguard Medical Associates, a multispecialty group practice in urban and suburban Boston, MA [15]. We enrolled 2128 mother–child pairs from the greater Boston area. We performed in person-study visits with participating mothers at the end of the first and second trimesters of pregnancy and with mothers and children after delivery and in infancy (median age 6.3 months), early childhood (median 3.2 years), mid childhood (median 7.7 years), early adolescence (median 12.9 years), and late adolescence (median 17.4 years). For this analysis, 1113 adolescents had data from early adolescent visit, 1013 of whom had non-missing BMI z-scores. As of March 2020, when in-person visits were paused due to the COVID pandemic, 619 had non-missing BMI z-scores at the late adolescent research visit, thus forming the analysis sample. Adolescents included and excluded from this analysis were comparable (Additional file 1: Table S1).

**Table 1:** Participant characteristics overall and according to overeating behavior^†^ at the Project Viva early adolescent visit

	Overall, n = 619	Early adolescent overeating behavior	
		Yes, n = 58 (9%)	No, n = 561 (91%)
**Mean (SD), median (IQR), or n (column %)**			
*Maternal characteristics*			
Age, years, median (IQR)	32.5 (6.2)	32.3 (7.4)	32.6 (6.0)
Pre-pregnancy BMI, kg/m^2^, mean (SD)	24.6 (5.2)	25.7 (6.6)	24.5 (5.0)
College graduate, n (%)			
No	162 (26%)	21 (36%)	141 (25%)
Yes	456 (74%)	37 (64%)	419 (75%)
Smoked during pregnancy, n (%)			
No	558 (90%)	51 (88%)	507 (91%)
Yes	60 (10%)	7 (12%)	53 (9%)
Excessive GWG, n (%)			
No	260 (43%)	27 (47%)	233 (42%)
Yes	349 (57%)	31 (53%)	318 (58%)
*Participant characteristics in early adolescence*			
Age, years, median (IQR)	12.9 (1.0)	13.0 (1.3)	12.9 (0.9)
Sex, n (%)			
Male	293 (47%)	20 (34%)	273 (49%)
Female	326 (53%)	38 (66%)	288 (51%)
Race/ethnicity, n (%)			
Black	92 (15%)	15 (26%)	77 (14%)
Hispanic	25 (4%)	6 (10%)	19 (3%)
White	400 (65%)	28 (48%)	372 (66%)
Other	101 (16%)	9 (16%)	92 (16%)
BMI z-score, units, mean (SD)	0.37 (1.08)	0.84 (0.95)	0.32 (1.08)
BMI category, n (%)			
Normal weight	451 (73%)	33 (57%)	418 (75%)
Overweight	91 (15%)	12 (21%)	79 (14%)
Obesity	77 (12%)	13 (22%)	64 (11%)
Pubertal development scale, points, mean (SD)	2.5 (0.8)	2.8 (0.8)	2.5 (0.8)
Overeating behavior (3-category exposure), n (%)			
No overeating	561 (91%)		561 (100%)
Overeating behavior without LOC	34 (5%)	34 (59%)	
Binge eating behavior (overeating with LOC)	24 (4%)	24 (41%)	

*BMI* body mass index, *GWG* gestational weight gain, *IQR* interquartile range, *LOC* loss of control

^†^Overeating behavior defined as a “yes” answer to: “In the past 12 months, have you ever eaten so much food in a short period of time that you would be embarrassed if others saw you?”

### Measures

#### Overeating and binge eating behavior

We assessed overeating and binge eating behavior during the early adolescent visit (median age 12.9 years), henceforth referred to as “baseline,” through the yes/no question on a self-administered questionnaire: “In the past 12 months, have you ever eaten so much food in a short period of time that you would be embarrassed if others saw you?” The question was adopted from the Questionnaire on Eating and Weight Patterns–Revised [16] and has test–retest reliability of 92% [17]. Those who responded affirmatively were asked a follow-up question about LOC: “During the times when you ate this way, did you feel you couldn’t stop eating or control what or how much you were eating?” This question has test–retest reliability of 84% [17].

For our primary analysis, we considered those who responded affirmatively to the first question to have “overeating behavior.” In secondary analyses, used a 3-category exposure. We categorized those who also endorsed LOC as having “binge eating behavior,” since overeating and LOC together comprise the clinical definition of binge eating. Those who responded positively to the first but not the second question had “overeating without LOC,” and we used “no overeating behavior” as the reference category.

#### Cardiometabolic outcomes

The study outcomes were collected at the late adolescent visit (median age 17.4 years), henceforth referred to as “follow-up.” Trained research assistants performed all anthropometric measurements.

At both study visits, we measured weight (Tanita scale model TBF-300A, Tanita Corporation of America, Arlington Heights, IL) and standing height (stadiometer, Shorr Productions, Olney MD) to calculate BMI and obtained age- and sex-specific BMI z-scores using Centers for Disease Control and Prevention (CDC) growth charts [18]. Per CDC references, we defined BMI < 85th percentile as normal weight, 85th to < 95th percentile as overweight, and ≥ 95th percentile as obesity. We measured waist circumference immediately superior to the iliac crest (Gulick II measuring tape, Performance Health, Warrenville, IL). We performed bioelectrical impedance analysis (BIA) (Tanita scale model TBF-300A, Tanita Corporation of America, Arlington Heights, IL) and dual-energy X-ray absorptiometry (DXA) scans (Hologic model Discovery A, Bedford, MA) to obtain body fat percentage. We measured seated systolic (SBP) and diastolic (DBP) blood pressures five times at one-minute intervals using calibrated automated oscillometric monitors (Dinamap Pro100, Tampa, Florida), and used mean blood pressure for analyses.

At follow-up, phlebotomists drew 8-h fasting blood samples. We assayed plasma glucose the same day, and the remaining assays were performed on samples stored at − 80 °C using the Roche Cobas C 501 Analyzer (Roche Diagnostics, Indianapolis, IN). We measured lipids (total cholesterol, high-density lipoprotein [HDL], and triglycerides, with enzymatic correction for glycerol), insulin, hemoglobin A1c (HbA1c), alanine aminotransferase (ALT), and high-sensitivity c-reactive protein (hsCRP) using the Roche Cobas 6000 system (Roche Diagnostics, Indianapolis, IN). We calculated the homeostatic model assessment-insulin resistance (HOMA-IR) as (glucose (mg/dL)*insulin (µU/mL))/405. We measured plasma leptin, adiponectin and interleukin-6 (IL-6) concentrations using ELISA assays (R&D Systems, Minneapolis, MN). Sample sizes for blood assay results ranged from n = 270–424 (for glucose).

#### Covariates

We identified potential covariates based on prior literature. Adolescent factors included sex, parent-reported race/ethnicity, and age at outcome measurement. We included race/ethnicity, a social construct, as a covariate as perceptions of race and experiences of racism can independently influence development, behaviors, and health. We considered parental factors as potential confounders given their potential genetic and environmental impact on eating pathologies, obesity, and metabolic syndrome. Maternal factors, collected at initial enrollment, included self-reported education, smoking habits, and pre-pregnancy weight and height, from which we calculated BMI. We calculated gestational weight gain (GWG) by subtracting pre-pregnancy weight from the last weight before delivery and classified excessive GWG per 2009 Institute of Medicine guidelines [19]. We additionally considered paternal BMI and also baseline pubertal stage assessed via the pubertal development scale [20], given its potential influence on eating pathology [21], obesity, and metabolic syndrome [22], but excluded these variables from models as neither influenced effect estimates.

### Statistical analyses

We used multivariable linear regression to examine associations of overeating behaviors (yes/no) with cardiometabolic outcomes, with adjustment for confounders. Because most biomarkers were right-skewed, we natural log-transformed all plasma measures. As a secondary analysis, we also conducted multivariable regressions using a 3-category exposure: no overeating behavior, overeating behavior without LOC, and overeating with LOC (or binge eating behavior). For all analyses, Model 1 was unadjusted. Model 2 adjusted for sex, race/ethnicity, and also follow-up age as a precision covariate since outcome distributions are expected to vary by age. Model 3 additionally adjusted for maternal factors: education status (college graduate vs. not), pre-pregnancy BMI (continuous), smoking during pregnancy (yes vs. no), and excessive GWG (vs. inadequate or adequate gain). Model 4 added adjustment for baseline BMI z-score. As BMI at follow-up may be an intermediate in the relationship between baseline eating behaviors and cardiometabolic outcomes, we examined potential mediation by BMI z-score at follow-up by including it as a covariate. We also ran models that adjusted for change in BMI z-score from baseline to follow-up instead of baseline BMI z-score.

A priori, we conducted analyses stratified by sex as eating behaviors and metabolic trends may differ by sex [21]. We also explored effect modification by pubertal stage, and baseline weight class (normal weight vs. overweight or obesity) using stratified models as well as including interaction terms in a combined model. We performed analyses with SAS Version 9.4 (Cary, NC).

## Results

### Demographics

Our study sample included 619 adolescents, with 326 females and 293 males; 65% of participants were White, 15% Black, 4% Hispanic, and 16% other races/ethnicities. Most participants had mothers with a college degree (74%) and mean maternal pre-pregnancy BMI was 24.6 kg/m^2^ (SD 5.2) (Table 1). At baseline, 73% of participants were normal weight, 15% overweight, and 12% had obesity; 58 (9%) reported overeating behavior, and of those, 24 (41%) endorsed LOC. Many characteristics between adolescents with and without overeating behavior were similar, including baseline age and pubertal score, as well as maternal age, smoking status, and GWG. Mean maternal pre-pregnancy BMI was higher in the group reporting overeating behavior (25.7 kg/m^2^) compared to those who did not (24.5 kg/m^2^). Adolescents reporting overeating behavior were also more likely to be female (66% vs. 51%), Hispanic (10% vs. 3%) or Black (26% vs. 14%), and have baseline overweight or obesity (43% vs. 25%) as compared to those without overeating behavior.

### Primary multivariable regression analyses

In multivariable regression models, early adolescent overeating behavior remained associated with adiposity, including a higher BMI (β = 1.72 kg/m^2^; 95% CI 0.43, 3.02), BMI z-score (β = 0.34 units; 95% CI 0.07, 0.61), waist circumference (β = 3.54 cm; 95% CI 0.26, 6.82), and total fat percentage by DXA (β = 4.03%; 95% CI 1.76, 6.31) after adjustment for sociodemographic and prenatal obesogenic factors (Table 2). Additional adjustment for early adolescent BMI z-score attenuated effect estimates to the null, except for total fat percentage by DXA, which remained independently associated with overeating behavior (β = 2.95%; 95% CI 1.03, 4.87).

**Table 2:** Cardiometabolic outcomes measured in late adolescence according to overeating behavior in early adolescence^†^, and multivariable regression models assessing the associations of overeating exposure with cardiometabolic outcomes in late adolescence

Late adolescent outcomes	N	Overeating behavior†		Effect estimates of overeating behavior on cardiometabolic outcomes in late adolescence			
		Yes, n = 58 (9%)	No, n = 561 (91%)	Model 1	Model 2	Model 3	Model 4
		*Mean (SD)*		*β (95% CI)*			
BMI, kg/m^2^	619	26.4 (6.8)	23.8 (5.1)	**2.61 (1.18, 4.03)**	**2.06 (0.64, 3.47)**	**1.72 (0.43, 3.02)**	0.46 (− 0.51, 1.43)
BMI z-score, units	619	0.82 (0.95)	0.36 (1.06)	**0.46 (0.17, 0.74)**	**0.40 (0.11, 0.68)**	**0.34 (0.07, 0.61)**	0.05 (− 0.13, 0.23)
Waist circumference, cm	618	85.7 (15.8)	81.3 (12.7)	**4.42 (0.89, 7.94)**	**4.28 (0.76, 7.81)**	**3.54 (0.26, 6.82)**	0.69 (− 1.96, 3.34)
Percent fat by BIA	616	26.8 (11.5)	21.0 (10.5)	**5.82 (2.94, 8.69)**	**3.17 (0.89, 5.45)**	**2.71 (0.57, 4.86)**	0.76 (− 0.92, 2.45)
Percent fat by DXA	428	33.3 (9.9)	27.7 (8.4)	5.65 (2.67, 8.63)	**4.29 (1.87, 6.71)**	**4.03 (1.76, 6.31)**	**2.95 (1.03, 4.87)**
Systolic BP, mmHg	615	109.4 (9.6)	111.1 (9.9)	− 1.72 (− 4.42, 0.99)	–0.17 (− 2.56, 2.21)	− 0.20 (− 2.58, 2.17)	− 0.76 (− 3.13, 1.60)
Diastolic BP, mmHg	615	65.4 (7.2)	64.5 (7.6)	0.95 (− 1.13, 3.02)	0.56 (− 1.54, 2.66)	0.49 (− 1.61, 2.58)	0.05 (− 2.04, 2.14)
		*Median (IQR)*		*Log-transformed outcomes*			
ALT, U/L	286	17.0 (8.0)	15.0 (6.0)	0.08 (− 0.08, 0.25)	0.11 (− 0.05, 0.28)	0.11 (− 0.06, 0.27)	0.09 (− 0.08, 0.25)
Total cholesterol, mg/dL	286	164.0 (43.0)	147.0 (42.0)	0.05 (− 0.03, 0.13)	0.03 (− 0.05, 0.12)	0.04 (− 0.04, 0.12)	0.04 (− 0.05, 0.12)
HDL, mg/dL	286	53.0 (19.0)	54.0 (15.0)	0.00 (− 0.10, 0.10)	− 0.02 (− 0.12, 0.07)	− 0.02 (− 0.11, 0.08)	− 0.01 (− 0.10, 0.09)
Triglycerides, mg/dL	285	66.8 (32.3)	60.0 (33.4)	0.02 (− 0.15, 0.18)	0.05 (− 0.13, 0.22)	0.05 (− 0.12, 0.22)	0.04 (− 0.13, 0.21)
IL-6, pg/mL	285	1.6 (2.5)	0.9 (0.8)	**0.54 (0.25, 0.83)**	**0.47 (0.17, 0.77)**	**0.44 (0.14, 0.75)**	**0.42 (0.12, 0.73)**
hsCRP, mg/L	285	0.7 (8.2)	0.4 (0.9)	**0.95 (0.31, 1.59)**	**0.79 (0.13, 1.45)**	**0.66 (0.01, 1.32)**	0.57 (− 0.07, 1.21)
Insulin, uU/ml	283	10.4 (11.6)	9.6 (7.6)	0.22 (− 0.02, 0.47)	0.15 (− 0.09, 0.40)	0.12 (− 0.13, 0.36)	0.09 (− 0.15, 0.32)
Glucose, mg/dL	424	85.5 (9.0)	84.0 (8.0)	0.02 (− 0.01, 0.04)	0.01 (− 0.01, 0.04)	0.01 (− 0.01, 0.04)	0.01 (− 0.01, 0.04)
HOMA-IR, units	270	2.1 (2.2)	1.9 (1.5)	**0.30 (0.05, 0.55)**	0.23 (− 0.03, 0.49)	0.18 (− 0.07, 0.44)	0.14 (− 0.11, 0.39)
HbA1c, percent	288	5.2 (0.4)	5.1 (0.4)	**0.03 (0.00, 0.05)**	0.02 (− 0.01, 0.04)	0.02 (− 0.01, 0.05)	0.02 (− 0.01, 0.05)
Leptin, ng/mL	285	11.6 (29.1)	8.8 (15.0)	**0.60 (0.05, 1.15)**	0.32 (− 0.11, 0.75)	0.28 (− 0.14, 0.70)	0.15 (− 0.21, 0.51)
Adiponectin, ug/mL	286	4.7 (3.3)	7.2 (4.6)	− **0.30 (**− **0.50, − 0.10)**	− **0.30 (**− **0.50, − 0.11)**	− **0.30 (**− **0.49, − 0.10)**	− **0.28 (**− **0.47, − 0.08)**

Model 1. Unadjusted

Model 2. Model 1 + adjustment for participant demographic factors (sex, race/ethnicity, age at late adolescent visit)

Model 3. Model 2 + maternal factors (education, pre-pregnancy BMI, smoked during pregnancy, and excessive GWG)

Model 4. Model 3 + participant BMI z-score score at early adolescent visit

*BMI* body mass index, *BIA* bioelectrical impedance analysis, *DXA* dual-energy X-ray absorptiometry, *BP* blood pressure, *IQR* interquartile range, *ALT* alanine aminotransferase, *HDL* high-density lipoprotein, *IL-6* interleukin-6, *hsCRP* high-sensitivity C-reactive protein, *HOMA-IR* homeostatic model assessment of insulin resistance, *HbA1c* hemoglobin A1c, *GWG* gestational weight gain

^†^Overeating behavior defined as a “yes” answer to: “In the past 12 months, have you ever eaten so much food in a short period of time that you would be embarrassed if others saw you?”

Bold text identifies those values for which the 95% CI does not include 0.0

Overeating behavior in early adolescence was positively associated with inflammatory markers after adjustment for sociodemographic and prenatal obesogenic factors (log-transformed IL-6 β = 0.44 pg/mL; 95% CI 0.14, 0.75, log-transformed hsCRP β = 0.66 mg/L; 95% CI 0.01, 1.32). In fully adjusted models, overeating behavior remained independently associated with IL-6 (log-transformed β = 0.42 pg/mL; 95% CI 0.12, 0.73), and the effect estimate for hsCRP remained positive, though the confidence interval crossed the null (log-transformed β = 0.57 mg/dL; 95% CI − 0.07, 1.21). These effect estimates represent a 53% difference in IL-6 and 77% difference in hsCRP between adolescents with and without overeating behavior. Regression models also demonstrated lower adiponectin in adolescents endorsing overeating behavior (log-transformed β = −0.28; 95% CI − 0.47, − 0.08) after full adjustment. Overeating behavior was initially associated with higher leptin levels in unadjusted models (log-transformed β = 0.60 ng/mL; 95% CI 0.05, 1.15); estimates remained directionally positive but attenuated with covariate adjustment.

Analyses of other cardiometabolic markers demonstrated few significant associations, though point estimates trended in expected directions. Overeating behavior was associated with higher HOMA-IR in the initial unadjusted model (log-transformed β = 0.30 units; 95% CI 0.05, 0.55), which remained positive but attenuated with adjustment. Across all models, there was little evidence of puberty impacting associations and no additional mediation by late adolescent BMI z-score after accounting for BMI z-score at baseline (Additional file 1: Table S2). There was also no meaningful difference when models were adjusted for change in BMI z-score from baseline to follow-up, rather than baseline BMI z-score (Additional file 1: Table S2). No strong evidence of effect modification by sex (Additional file 1: Table S4) or baseline weight class (Additional file 1: Table S5) was seen.

### Secondary multivariable regression analyses

Adolescents reporting binge eating behavior, i.e., overeating with LOC, generally had the greatest adiposity as compared to those with overeating without loss of control and those without overeating (Table 3, Additional file 1: Table S3). While effect estimates remained directionally positive, the only persistent association with adiposity after adjustment for baseline BMI z-score was between the subgroup with binge eating behavior and body fat by DXA (β = 3.47%; 95% CI 0.73, 6.21). Compared to those who reported no overeating behavior, binge eating behavior was associated with higher IL-6 and lower adiponectin even after adjusting for baseline BMI z-score (log-transformed IL-6 β = 0.53 pg./ml; 95% CI 0.13, 0.93, log-transformed adiponectin β = −0.40 ug/ml; 95% CI − 0.66, − 0.14). These estimates represent 70% higher IL-6 and 33% lower adiponectin compared to those without overeating behavior whereas effect sizes were smaller and crossed the null in adolescents without LOC. Leptin trended positively with highest levels in those reporting binge eating behavior compared to those endorsing overeating without LOC, but confidence intervals included the null.

**Table 3:** Multivariable regression models assessing the associations of overeating behavior in early adolescence, further categorized as binge eating behavior (overeating with loss of control (LOC)^†^ and overeating behavior without LOC),^††^ with cardiometabolic outcomes in late adolescence among adolescents in the Project Viva cohort

Late adolescent outcomes	3-category exposure	Effect estimates of binge eating behavior and overeating behavior without LOC on cardiometabolic outcomes in late adolescence			
		Model 1	Model 2	Model 3	Model 4
		*β (95% CI)*			
BMI, kg/m^2^	Binge eating behavior	**3.50 (1.35, 5.65)**	**2.93 (0.82, 5.04)**	**2.46 (0.53, 4.39)**	0.88 (− 0.56, 2.33)
	Overeating behavior without LOC	**1.98 (0.15, 3.80)**	1.42 (− 0.39, 3.24)	1.19 (− 0.48, 2.85)	0.15 (− 1.09, 1.39)
	No overeating behavior	0.0 (ref)	0.0 (ref)	0.0 (ref)	0.0 (ref)
BMI z-score, units	Binge eating behavior	**0.58 (0.15, 1.01)**	**0.48 (0.06, 0.91)**	0.39 (− 0.01, 0.79)	0.03 (− 0.24, 0.30)
	Overeating behavior without LOC	**0.37 (0.01, 0.73)**	0.33 (− 0.03, 0.70)	0.30 (− 0.04, 0.65)	0.07 (− 0.17, 0.30)
	No overeating behavior	0.0 (ref)	0.0 (ref)	0.0 (ref)	0.0 (ref)
Waist circumference, cm	Binge eating behavior	**7.94 (2.62, 13.26)**	**7.30 (2.05, 12.54)**	**6.22 (1.34, 11.11)**	2.67 (− 1.27, 6.61)
	Overeating behavior without LOC	1.93 (− 2.58, 6.43)	2.10 (− 2.42, 6.61)	1.59 (− 2.62, 5.80)	− 0.75 (− 4.13, 2.64)
	No binge eating behavior	0.0 (ref)	0.0 (ref)	0.0 (ref)	0.0 (ref)
Percent fat by BIA	Binge eating behavior	**4.87 (0.53, 9.22)**	**3.72 (0.32, 7.12)**	**3.11 (**− **0.08, 6.31)**	0.69 (− 1.82, 3.20)
	Overeating behavior without LOC	**6.48 (2.80, 10.17)**	2.77 (− 0.16, 5.70)	2.42 (− 0.33, 5.18)	0.82 (− 1.34, 2.98)
	No overeating behavior	0.0 (ref)	0.0 (ref)	0.0 (ref)	0.0 (ref)
Percent fat by DXA	Binge eating behavior	**6.31 (2.05, 10.57)**	**5.79 (2.37, 9.22)**	**5.00 (1.74, 8.25)**	**3.47 (0.73, 6.21)**
	Overeating behavior without LOC	**5.07 (1.04, 9.09)**	**2.95 (**− **0.29, 6.19)**	**3.19 (0.13, 6.25)**	2.50 (− 0.07, 5.07)
	No overeating behavior	0.0 (ref)	0.0 (ref)	0.0 (ref)	0.0 (ref)
Systolic BP, mmHg	Binge eating behavior	0.52 (− 3.53, 4.57)	1.23 (− 2.29, 4.76)	1.14 (− 2.37, 4.66)	0.46 (− 3.03, 3.95)
	Overeating behavior without LOC	− 3.34 (− 6.83, 0.14)	− 1.23 (− 4.31, 1.85)	− 1.21 (− 4.28, 1.86)	− 1.67 (− 4.71, 1.37)
	No overeating behavior	0.0 (ref)	0.0 (ref)	0.0 (ref)	0.0 (ref)
Diastolic BP, mmHg	Binge eating behavior	2.01 (− 1.11, 5.12)	2.11 (− 0.99, 5.22)	2.00 (− 1.10, 5.09)	1.47 (− 1.61, 4.55)
	Overeating behavior without LOC	0.18 (− 2.50, 2.85)	− 0.61 (− 3.32, 2.10)	− 0.64 (− 3.35, 2.06)	− 1.00 (− 3.68, 1.68)
	No overeating behavior	0.0 (ref)	0.0 (ref)	0.0 (ref)	0.0 (ref)
	*Log-transformed outcomes*				
ALT, U/L	Binge eating behavior	0.14 (− 0.09, 0.36)	0.15 (− 0.07, 0.37)	0.14 (− 0.09, 0.36)	0.13 (− 0.09, 0.35)
	Overeating behavior without LOC	0.03 (− 0.21, 0.26)	0.07 (− 0.16, 0.30)	0.07 (− 0.16, 0.31)	0.05 (− 0.18, 0.28)
	No overeating behavior	0.0 (ref)	0.0 (ref)	0.0 (ref)	0.0 (ref)
Total cholesterol, mg/dL	Binge eating behavior	0.08 (− 0.03, 0.19)	0.07 (− 0.04, 0.18)	0.09 (− 0.02, 0.20)	0.09 (− 0.03, 0.20)
	Overeating behavior without LOC	0.02 (− 0.10, 0.13)	− 0.01 (− 0.13, 0.11)	− 0.01 (− 0.13, 0.11)	− 0.02 (− 0.14, 0.10)
	No overeating behavior	0.0 (ref)	0.0 (ref)	0.0 (ref)	0.0 (ref)
HDL, mg/dL	Binge eating behavior	− 0.01 (− 0.15, 0.12)	− 0.03 (− 0.15, 0.10)	− 0.01 (− 0.13, 0.12)	0.00 (− 0.12, 0.12)
	Overeating behavior without LOC	0.02 (− 0.12, 0.16)	− 0.02 (− 0.16, 0.12)	− 0.03 (− 0.16, 0.10)	− 0.01 (− 0.14, 0.12)
	No overeating behavior	0.0 (ref)	0.0 (ref)	0.0 (ref)	0.0 (ref)
Triglycerides, mg/dL	Binge eating behavior	0.06 (− 0.17, 0.29)	0.09 (− 0.14, 0.33)	0.09 (− 0.14, 0.33)	0.09 (− 0.15, 0.32)
	Overeating behavior without LOC	− 0.02 (− 0.26, 0.21)	0.00 (− 0.24, 0.24)	0.01 (− 0.23, 0.24)	− 0.01 (− 0.24, 0.23)
	No overeating behavior	0.0 (ref)	0.0 (ref)	0.0 (ref)	0.0 (ref)
IL-6, pg/mL	Binge eating behavior	**0.63 (0.24, 1.03)**	**0.60 (0.20, 0.99)**	**0.54 (0.14, 0.95)**	**0.53 (0.13, 0.93)**
	Overeating behavior without LOC	**0.43 (0.02, 0.85)**	0.33 (− 0.09, 0.75)	0.33 (− 0.09, 0.76)	0.30 (− 0.12, 0.73)
	No overeating behavior	0.0 (ref)	0.0 (ref)	0.0 (ref)	0.0 (ref)
hsCRP, mg/L	Binge eating behavior	0.69 (− 0.18, 1.56)	0.58 (− 0.30, 1.46)	0.30 (− 0.57, 1.18)	0.25 (− 0.60, 1.10)
	Overeating behavior without LOC	**1.24 (0.33, 2.14)**	**1.02 (0.08, 1.96)**	**1.06 (0.15, 1.98)**	**0.93 (0.04, 1.83)**
	No overeating behavior	0.0 (ref)	0.0 (ref)	0.0 (ref)	0.0 (ref)
Insulin, uU/ml	Binge eating behavior	**0.36 (0.02, 0.69)**	0.31 (− 0.02, 0.65)	0.24 (− 0.09, 0.57)	0.23 (− 0.09, 0.55)
	Overeating behavior without LOC	0.09 (− 0.24, 0.42)	− 0.02 (− 0.36, 0.33)	− 0.01 (− 0.35, 0.33)	− 0.06 (− 0.39, 0.27)
	No overeating behavior	0.0 (ref)	0.0 (ref)	0.0 (ref)	0.0 (ref)
Glucose, mg/dL	Binge eating behavior	0.03 (− 0.01, 0.07)	0.03 (− 0.01, 0.07)	0.03 (− 0.01, 0.07)	0.03 (− 0.01, 0.07)
	Overeating behavior without LOC	0.00 (− 0.03, 0.04)	0.00 (− 0.03, 0.04)	0.00 (− 0.03, 0.04)	0.00 (− 0.03, 0.04)
	No overeating behavior	0.0 (ref)	0.0 (ref)	0.0 (ref)	0.0 (ref)
HOMA-IR, units	Binge eating behavior	**0.49 (0.13, 0.84)**	**0.44 (0.08, 0.80)**	0.34 (− 0.01, 0.70)	0.32 (− 0.03, 0.66)
	Overeating behavior without LOC	0.13 (− 0.21, 0.47)	0.02 (− 0.33, 0.37)	0.04 (− 0.31, 0.38)	− 0.03 (− 0.36, 0.31)
	No overeating behavior	0.0 (ref)	0.0 (ref)	0.0 (ref)	0.0 (ref)
HbA1c, percent	Binge eating behavior	**0.03 (0.00, 0.07)**	0.03 (− 0.01, 0.06)	0.03 (0.00, 0.07)	0.03 (0.00, 0.07)
	Overeating behavior without LOC	0.02 (− 0.02, 0.06)	0.01 (− 0.03, 0.05)	0.01 (− 0.03, 0.05)	0.01 (− 0.03, 0.05)
	No overeating behavior	0.0 (ref)	0.0 (ref)	0.0 (ref)	0.0 (ref)
Leptin, ng/mL	Binge eating behavior	**0.78 (0.04, 1.51)**	**0.59 (0.03, 1.15)**	0.42 (− 0.13, 0.97)	0.34 (− 0.13, 0.81)
	Overeating behavior without LOC	0.39 (− 0.41, 1.19)	− 0.03 (− 0.65, 0.60)	0.11 (− 0.50, 0.71)	− 0.09 (− 0.61, 0.43)
	No overeating behavior	0.0 (ref)	0.0 (ref)	0.0 (ref)	0.0 (ref)
Adiponectin, ug/mL	Binge eating behavior	− **0.44 (**− **0.72,** − **0.16)**	− **0.43 (**− **0.69,** − **0.17)**	− **0.41 (**− **0.68,** − **0.15)**	− **0.40 (**− **0.66,** − **0.14)**
	Overeating behavior without LOC	− 0.15 (− 0.43, 0.14)	− 0.16 (− 0.43, 0.12)	− 0.17 (− 0.44, 0.11)	− 0.14 (− 0.41, 0.14)
	No overeating behavior	0.0 (ref)	0.0 (ref)	0.0 (ref)	0.0 (ref)

Model 1. Unadjusted

Model 2. Model 1 + adjustment for participant factors (sex, race/ethnicity, age at late adolescent visit)

Model 3. Model 2 + maternal factors (education, pre-pregnancy BMI, smoked during pregnancy, and excessive GWG)

Model 4. Model 3 + participant BMI z-score at early adolescent visit

*LOC* loss of control, *BMI* body mass index, *BIA* bioelectrical impedance analysis, *DXA* dual-energy X-ray absorptiometry, *BP* blood pressure, *ALT* alanine aminotransferase, *HDL* high-density lipoprotein, *IL-6* interleukin-6, *hsCRP* high-sensitivity C-reactive protein, *HOMA-IR* homeostatic model assessment of insulin resistance, *HbA1c* hemoglobin A1c, *GWG* gestational weight gain

^†^Binge eating behavior defined as a “yes” answer to: “In the past 12 months, have you ever eaten so much food in a short period of time that you would be embarrassed if others saw you?” AND “yes” to: “During the times when you ate this way, did you feel you couldn't stop eating or control what or how much you were eating?”

^††^Overeating behavior without LOC defined as “no” response to the second question

Bold text identifies those values for which the 95% CI does not include 0.0

In unadjusted models, binge eating behavior was positively associated with HOMA-IR (log-transformed β = 0.49 units; 95% CI 0.13, 0.84), insulin (log-transformed β = 0.36 uU/mL; 95% CI 0.02, 0.69), and HbA1c (log-transformed β = 0.03%; 95% CI 0.00, 0.07), all of which attenuated after covariate adjustment. No significant differences in blood pressure, liver function, or lipid measures were demonstrated between groups.

## Discussion

In a general population sample of over 600 adolescents, we found that adolescents endorsing overeating behavior had higher adiposity than their peers approximately five years later. Adolescents reporting overeating behavior also had poorer inflammatory and adipokine profiles, most notably by measures of IL-6 and adiponectin, but no consistent differences in other markers of cardiometabolic risk. These associations were only partially accounted for by higher baseline BMI. Additionally, binge eating behavior demonstrated more durable associations than overeating without LOC, likely because greater effect size magnitude outweighed the smaller number of those with the exposure behavior. Overall, our study suggests that overeating and binge eating behavior is associated with longitudinal changes in physiology that increase cardiovascular risk, with some, but not all, associations independent of adiposity.

Our results expand existing literature on the association between disordered eating and adiposity, finding that adolescents reporting overeating behaviors had greater adiposity than those who did not, and that binge eating conferred higher risk of adiposity than overeating without LOC [2, 13]. While associations between overeating without LOC and adiposity trended in a positive direction, it did not reach significance after adjustment for confounders and early adolescence BMI, similar to prior research [13]. However, besides an association with body fat by DXA, we found minimal evidence suggesting excess future weight gain in those endorsing overeating or binge eating. This contrasts with results from a study of youth with overweight/obesity endorsing overeating with LOC who had excess weight gain [10], but aligns with two prospective studies of girls of all weights where binge eating did not predict extra weight gain [23] or greater odds of new-onset obesity [24]. These variations could be explained by participant characteristics—in our study, disordered eating was defined differently, a majority of participants had normal weight, and those with overeating and binge eating behavior were predominantly female.

Several mechanisms can explain the higher prevalence of baseline overweight and obesity *without* evidence of subsequent excess weight gain in those reporting overeating behavior. First, our exposure question was designed to capture disordered eating behavior during the previous year, so it is possible that BMI measured at the early adolescent visit was more a mediator than a confounder. Second, our exposure and reference groups likely had both intragroup heterogeneity and intergroup similarity: the group endorsing overeating behavior comprised individuals with varied severity, including some who may have remitted, while the reference group conceivably included those who initiated overeating behavior after exposure assessment. Third, a subset of adolescents who endorsed overeating behavior may have considered a normal caloric intake inappropriate due to social pressures towards thinness, such that they endorsed embarrassment but were weight-stable. Regardless, as overeating behavior in our study was associated with a 0.34 SD higher BMI z-score and no normalization of weight over time, and evidence-based guidelines suggest that z-score reductions of even 0.15 units can improve cardiovascular health in youth with overweight or obesity [25], further research should clarify the psychological experiences of these adolescents and their associations with physical health over time.

We also confirmed our hypothesis that overeating behavior was prospectively associated with higher systemic inflammation as reflected by IL-6, which has been of clinical interest in predicting cardiovascular disease [26]. While some studies have found links between eating disorders and higher IL-6 [27], no studies to our knowledge have prospectively examined associations of overeating behavior and IL-6. Meanwhile, the influence of adiposity on higher hsCRP has previously been reported [28], and our data replicated a pediatric study associating binge eating with higher hsCRP that was dependent on adiposity [28] while differing from an adult study that reported an independent relationship [5]. Why overeating and inflammation are associated is uncertain; one hypothesis is that those endorsing overeating behavior may have also participated in dieting, leading to weight variability—but overall a stable BMI z-score—and weight variability has been shown to be associated with higher inflammation [29]. Given the health implications of longstanding systemic inflammation, further clarification on how overeating behavior play a role in inflammation is key.

Adiponectin is an anti-inflammatory adipokine that is inversely related with adiposity [7], but has been minimally studied in eating disorders. One previous study found lower adiponectin in women with binge eating disorder that poorly correlated with binge frequency, but did not assess adiposity as a potential confounder [30]. We demonstrated independent associations of even potentially infrequent overeating events with lower adiponectin independent of adiposity, and expanded the literature of adipokine dysfunction to adolescents. Our leptin findings, however, did not consistently align with hypotheses that overeating would be associated with higher leptin levels. One possible explanation is that leptin secretion can be temporarily influenced by recent overeating episodes or binges, as one study demonstrated that participants who binged the prior evening had blunted leptin secretion on morning blood draws [31]. It is also possible that our study differed in statistical approach from previous literature—we log-transformed our right-skewed dataset to improve normality whereas several existing studies did not [31, 32].

Finally, we did not find significant associations between adolescent overeating or binge eating behavior and other cardiometabolic markers, including lipid levels, blood pressure, and liver function. These findings differed from adult studies [3, 4] and a prospective study of LOC eating in children with personal or familial risk for obesity [11] that associated binge eating with poorer cardiometabolic profiles and triglyceride levels. One explanation may have been inclusion criteria: we studied a community-based sample of adolescents with a range of overeating and binge eating and varying baseline cardiometabolic risk. Overall, this finding is hopeful: despite adiposity, inflammation, and adipokine dysregulation being harbingers of future cardiovascular disease [33, 34], adolescents endorsing overeating or binge eating behavior have not yet shown signs of cardiometabolic injury.

Study limitations included using a study sample that was slightly different from the original longitudinal cohort due to loss to follow-up. While this can introduce bias, loss to follow-up is a common challenge in longitudinal studies. Second, our exposures were defined using two nested self-reported questions, which unlike comprehensive eating disorder questionnaires or behavioral assessments by trained assistants or clinicians, optimized for response rates and minimal attrition over our prospective study, rather than for the sensitivity or specificity of the definitions. Previous research has also identified that assessing overeating and binge eating behaviors among adolescents can be challenging due to the vagueness of terms such as “loss of control” [35, 36]. While our measure aimed to reduce this vagueness by defining loss of control, i.e., “couldn’t stop eating,” our self-report measure may have resulted in some misclassification due to misinterpretation by respondents. Additionally, our primary exposure included but was not powered to primarily interrogate for binge eating. However, we focused on assessing overeating behaviors, more prevalent in the adolescent population, while performing secondary analyses for overeating with LOC, i.e., binge eating. We also did not ascertain the length of exposure or age of eating behavior initiation, but prior studies using validated questionnaires similarly assessed for prior behaviors over a distinct period, and often did not assess specific age of onset, which is fundamentally challenging to determine in a behavior that may evolve gradually and may be impacted by recall bias. We did not have a measure of physical fitness, which might be a confounder of the relationship between binge eating behaviors and cardiometabolic health. Lastly, we ran separate statistical models for each outcome. However, we noted high correlations within outcomes that should trend together—for example, between adiposity measures—and conservatively interpreted isolated findings.

These limitations were balanced by several strengths. First, we examined multiple components of metabolic syndrome, adiposity, and inflammation, unlike the narrower scopes of previous studies. We also treated these outcomes continuously rather than as categorical variables, given our broad sample of generally healthy adolescents. Third, we utilized a longitudinal cohort with extensive data collected from study participants and their mothers, allowing for robust confounding adjustment. Lastly, we examined the impact of overeating and binge eating behavior across weight groups, including predominantly adolescents with normal weight who may be phenotypically different from more heavily studied peers with higher BMIs.

## Conclusions

In conclusion, early adolescents reporting overeating and binge eating behaviors had higher adiposity and poorer inflammatory and adipokine profiles than their peers in late adolescence but did not differ in other risk markers. These associations were in part independent of adiposity. Further research should explore whether overeating and binge eating behavior itself or closely associated psychosocial factors are upstream of these physiological outcomes. Clinically, studies have reassuringly shown that treatment can improve both disordered eating behaviors and physiologic profiles [37], so in addition to striving to address upstream factors such as caloric restriction and diet culture and that place adolescents at higher at risk for overeating behaviors, screening for overeating behavior and loss of control, especially in adolescents with overweight or obesity, can allow for early intervention. Adolescence may be a key time period during which encouraging a body inclusive environment and promoting healthy eating behaviors can prevent the development of cardiometabolic disease.

## Supplementary Information

**Additional file 1. Table S1**: Characteristics of included vs. excluded participants. **Table S2**: Multivariable regression models assessing the associations of overeating exposure with cardiometabolic outcomes in late adolescence, with adjustment for BMI z-score at different timepoints. **Table S3**: Multivariable regression models assessing the associations of overeating behavior in early adolescence, further categorized as binge eating behavior (overeating with loss of control^†^ and overeating without LOC [reference category]),^††^ with cardiometabolic outcomes in late adolescence among adolescents in the Project Viva cohort. **Table S4**: Multivariable regression models assessing the associations of overeating behavior in early adolescence, with cardiometabolic outcomes in late adolescence among adolescents in the Project Viva cohort, stratified by sex at birth. **Table S5**: Multivariable regression models assessing the associations of overeating behavior† in early adolescence, with cardiometabolic outcomes in late adolescence among adolescents in the Project Viva cohort, stratified by body mass index category in early adolescence.

## Data Availability

The datasets generated during and/or analyzed during the current study are not publicly available because informed consent provisions did not cover public data sharing, but are available from the corresponding author on reasonable request.

## Abbreviations

BMI: Body mass index
CI: Confidence interval
LOC: Loss of control
HDL: High-density lipoprotein
CDC: Centers for Disease Control and Prevention
BIA: Bioelectric impedance analysis
DXA: Dual-energy X-ray absorptiometry
SBP: Systolic blood pressure
DBP: Diastolic blood pressure
LDL: Low-density lipoprotein
HbA1c: Hemoglobin A1c
HOMA-IR: Homeostatic model assessment-insulin resistance
IL-6: Interleukin-6
ELISA: Ultrasensitive enzyme-linked immunosorbent assay
ALT: Alanine aminotransferase
hsCRP: High-sensitivity c-reactive protein
SD: Standard deviation
IQR: Interquartile range
GWG: Gestational weight gain
