# Supplementary material for: Adolescent overeating and binge eating behavior in relation to subsequent cardiometabolic risk outcomes: a prospective cohort study
Source: J Eat Disord. 2022 Sep 13;10:140. doi: 10.1186/s40337-022-00660-4 (PMC9472357; doi:10.1186/s40337-022-00660-4)
Supplement: Supplementary file 1 — Additional file 1. Table S1: Characteristics of included vs. excluded participants. Table S2: Multivariable regression models assessing the associations of overeating exposure with cardiometabolic outcomes in late adolescence, with adjustment for BMI z-score at different timepoints. Table S3: Multivariable regression models assessing the associations of overeating behavior in early adolescence, further categorized as binge eating behavior (overeating with loss of control† and overeating without LOC [reference category]),†† with cardiometabolic outcomes in late adolescence among adolescents in the Project Viva cohort. Table S4: Multivariable regression models assessing the associations of overeating behavior in early adolescence, with cardiometabolic outcomes in late adolescence among adolescents in the Project Viva cohort, stratified by sex at birth. Table S5: Multivariable regression models assessing the associations of overeating behavior† in early adolescence, with cardiometabolic outcomes in late adolescence among adolescents in the Project Viva cohort, stratified by body mass index category in early adolescence. [file 40337_2022_660_MOESM1_ESM.docx]

**Adolescent overeating and binge eating behavior in relation to subsequent cardiometabolic risk outcomes: a prospective cohort study**

**Supplemental Tables**

**Supplemental Table 1.** Characteristics of included vs. excluded participants

**Supplemental Table 2**: Multivariable regression models assessing the associations of overeating exposure with cardiometabolic outcomes in late adolescence, with adjustment for BMI z-score at different timepoints

**Supplemental Table 3.** Multivariable regression models assessing the associations of overeating behavior in early adolescence, further categorized as binge eating behavior (overeating with loss of control**^†^** and overeating without LOC [reference category]),**^††^** with cardiometabolic outcomes in late adolescence among adolescents in the Project Viva cohort

**Supplemental Table 4.** Multivariable regression models assessing the associations of overeating behavior in early adolescence, with cardiometabolic outcomes in late adolescence among adolescents in the Project Viva cohort, stratified by sex at birth

**Supplemental Table 5.** Multivariable regression models assessing the associations of overeating behavior† in early adolescence, with cardiometabolic outcomes in late adolescence among adolescents in the Project Viva cohort, stratified by body mass index category in early adolescence.

**Supplemental Table 1.** Characteristics of included vs. excluded participants

|  | **Among 2128 Project Viva live births** | | **P-value** |
| --- | --- | --- | --- |
|  | **Included,** n=619 | **Excluded,** n=1509 |  |
| ***Maternal characteristics*** | *n (column %), mean (SD), or median (IQR)* | |  |
| **Age, years,** median (IQR) | 32.5 (6.2) | 32.0 (6.4) | 0.0002 |
| **Pre-pregnancy BMI, kg/m^2^,** mean (SD) | 24.6 (5.2) | 25.0 (5.7) | 0.14 |
| **College graduate,** n (%) | | | <.0001 |
| No | 162 (26%) | 582 (39%) |  |
| Yes | 456 (74%) | 904 (61%) |  |
| **Smoked during pregnancy,** n (%) | | | 0.01 |
| No | 558 (90%) | 1283 (86%) |  |
| Yes | 60 (10%) | 206 (14%) |  |
| **Excessive gestational weight gain,** n (%) | | | 0.68 |
| No | 260 (43%) | 607 (42%) |  |
| Yes | 349 (57%) | 848 (58%) |  |
| ***Participant characteristics in early adolescence*** | | | |
| **Age, years,** median (IQR) | 13.1 (0.9) | 13.4 (1.1) | <.0001 |
| **Sex,** n (%) | | | 0.01 |
| Male | 293 (47%) | 803 (53%) |  |
| Female | 326 (53%) | 706 (47%) |  |
| **Race/ethnicity,** n (%) | | | 0.004 |
| Black | 92 (15%) | 263 (18%) |  |
| Hispanic | 25 (4%) | 103 (7%) |  |
| White | 400 (65%) | 942 (63%) |  |
| Other | 101 (16%) | 183 (12%) |  |
| **BMI z-score, units,** mean (SD) | 0.37 (1.08) | 0.39 (1.04) | 0.77 |
| **BMI category,** n (%) | | | 0.98 |
| Normal weight | 451 (73%) | 301 (73%) |  |
| Overweight | 91 (15%) | 63 (15%) |  |
| Obesity | 77 (12%) | 51 (12%) |  |
| **Pubertal development scale, points,** mean (SD) | 2.5 (0.8) | 2.6 (0.8) | 0.02 |
| **Overeating behavior**† **(3-category exposure),** n (%) | | | 0.43 |
| No overeating | 561 (91%) | 457 (93%) |  |
| Overeating behavior without LOC | 34 (5%) | 19 (4%) |  |
| Binge eating behavior (overeating with LOC) | 24 (4%) | 18 (4%) |  |

†Overeating behavior defined as a “yes” answer to: “In the past 12 months, have you ever eaten so much food in a short period of time that you would be embarrassed if others saw you?”

BMI = body mass index; LOC = loss of control

**Supplemental Table 2**: Multivariable regression models assessing the associations of overeating exposure with cardiometabolic outcomes in late adolescence, with adjustment for BMI z-score at different timepoints

|  | Effect estimates of overeating behavior on cardiometabolic outcomes in late adolescence | | |
| --- | --- | --- | --- |
| **Late adolescent outcomes** | **Model 4** | **Model 5** | **Model 6** |
|  | ß (95% CI) | | |
| BMI, kg/m^2^ | 0.46 (-0.51, 1.43) | -- | **1.82 (0.59, 3.05)** |
| BMI z-score, units | 0.05 (-0.13, 0.23) | -- | **0.37 (0.12, 0.61)** |
| Waist circumference, cm | 0.69 (-1.96, 3.34) | -- | **3.74 (0.56, 6.92)** |
| Percent fat by BIA | 0.76 (-0.92, 2.45) | -- | **2.88 (0.84, 4.92)** |
| Percent fat by DXA | **2.95 (1.03, 4.87)** | -- | **3.75 (1.53, 5.97)** |
| Systolic BP, mmHg | -0.76 (-3.13, 1.60) | -0.87 (-3.18, 1.44) | -0.11 (-2.47, 2.25) |
| Diastolic BP, mmHg | 0.05 (-2.04, 2.14) | -0.03 (-2.09, 2.03) | 0.55 (-1.53, 2.64) |
| **Log-transformed outcomes** |  |  |  |
| ALT, U/L | 0.09 (-0.08, 0.25) | 0.07 (-0.09, 0.23) | 0.10 (-0.06, 0.27) |
| Total cholesterol, mg/dL | 0.04 (-0.05, 0.12) | 0.03 (-0.05, 0.12) | 0.04 (-0.04, 0.12) |
| HDL, mg/dL | -0.01 (-0.10, 0.09) | 0.00 (-0.09, 0.09) | -0.02 (-0.11, 0.08) |
| Triglycerides, mg/dL | 0.04 (-0.13, 0.21) | 0.04 (-0.13, 0.21) | 0.05 (-0.12, 0.22) |
| IL-6, pg/mL | **0.42 (0.12, 0.73)** | **0.41 (0.11, 0.71)** | **0.44 (0.14, 0.74)** |
| hsCRP, mg/L | 0.57 (-0.07, 1.21) | 0.49 (-0.12, 1.10) | 0.64 (-0.01, 1.28) |
| Insulin, uU/ml | 0.09 (-0.15, 0.32) | 0.04 (-0.18, 0.27) | 0.10 (-0.14, 0.34) |
| Glucose, mg/dL | 0.01 (-0.01, 0.04) | 0.01 (-0.01, 0.04) | 0.02 (-0.01, 0.04) |
| HOMA-IR, units | 0.14 (-0.11, 0.39) | 0.09 (-0.14, 0.33) | 0.17 (-0.08, 0.42) |
| HbA1c, percent | 0.02 (-0.01, 0.05) | 0.02 (-0.01, 0.05) | 0.02 (-0.01, 0.05) |
| Leptin, ng/mL | 0.15 (-0.21, 0.51) | 0.07 (-0.24, 0.39) | 0.26 (-0.15, 0.67) |
| Adiponectin, ug/mL | **-0.28 (-0.47, -0.08)** | **-0.25 (-0.44, -0.06)** | **-0.29 (-0.48, -0.09)** |

**Model 4**. Adjusted for participant demographic factors (sex, race/ethnicity, age at mid-adolescent visit) + maternal factors (education, pre-pregnancy BMI, smoked during pregnancy, and excessive GWG) **+ participant BMI z-score at early adolescent visit**

**Model 5**. Adjusted for participant demographic factors (sex, race/ethnicity, age at mid-adolescent visit) + maternal factors (education, pre-pregnancy BMI, smoked during pregnancy, and excessive GWG) **+ participant BMI z-score at early adolescent visit + BMI z-score at late adolescent visit**

**Model 6**. Adjusted for participant demographic factors (sex, race/ethnicity, age at mid-adolescent visit) + maternal factors (education, pre-pregnancy BMI, smoked during pregnancy, and excessive GWG) **+ change in BMI z-score from early adolescent to late adolescent visit**

**Supplemental Table 3.** Multivariable regression models assessing the associations of overeating behavior in early adolescence, further categorized as binge eating behavior (overeating with loss of control**^†^** and overeating without LOC [reference category]),**^††^** with cardiometabolic outcomes in late adolescence among adolescents in the Project Viva cohort

|  | **3-category exposure** | **Effect estimates of binge eating on cardiometabolic outcomes at late adolescence** | | | |
| --- | --- | --- | --- | --- | --- |
|  |  | **Model 1** | **Model 2** | **Model 3** | **Model 4** |
|  |  | ***ß (95% CI)*** | | | |
| ***Late adolescent outcomes*** |  |  |  |  |  |
| BMI, kg/m^2^ | |  |  |  |  |
|  | Binge eating behavior | 1.53 (-1.22, 4.27) | 1.51 (-1.19, 4.20) | 1.27 (-1.20, 3.75) | 0.74 (-1.11, 2.58) |
|  | Overeating behavior without LOC | 0.0 (ref) | 0.0 (ref) | 0.0 (ref) | 0.0 (ref) |
|  | No overeating behavior | **-1.98 (-3.80, -0.15)** | -1.42 (-3.24, 0.39) | -1.19 (-2.85, 0.48) | -0.15 (-1.39, 1.09) |
| BMI z-score, units | |  |  |  |  |
|  | Binge eating behavior | 0.21 (-0.34, 0.76) | 0.15 (-0.39, 0.70) | 0.09 (-0.42, 0.60) | -0.03 (-0.38, 0.31) |
|  | Overeating behavior without LOC | 0.0 (ref) | 0.0 (ref) | 0.0 (ref) | 0.0 (ref) |
|  | No overeating behavior | **-0.37 (-0.73, -0.01)** | -0.33 (-0.70, 0.03) | -0.30 (-0.65, 0.04) | -0.07 (-0.30, 0.17) |
| Waist circumference, cm | |  |  |  |  |
|  | Binge eating behavior | 6.02 (-0.79,12.82) | 5.20 (-1.51,11.92) | 4.64 (-1.63,10.90) | 3.42 (-1.61, 8.44) |
|  | Overeating behavior without LOC | 0.0 (ref) | 0.0 (ref) | 0.0 (ref) | 0.0 (ref) |
|  | No overeating behavior | -1.93 (-6.43, 2.58) | -2.10 (-6.61, 2.42) | -1.59 (-5.80, 2.62) | 0.75 (-2.64, 4.13) |
| Percent fat by BIA | |  |  |  |  |
|  | Binge eating behavior | -1.61 (-7.17, 3.95) | 0.95 (-3.41, 5.30) | 0.69 (-3.41, 4.78) | -0.13 (-3.34, 3.07) |
|  | Overeating behavior without LOC | 0.0 (ref) | 0.0 (ref) | 0.0 (ref) | 0.0 (ref) |
|  | No overeating behavior | **-6.48 (-10.2, -2.80)** | -2.77 (-5.70, 0.16) | -2.42 (-5.18, 0.33) | -0.82 (-2.98, 1.34) |
| Percent fat by DXA | |  |  |  |  |
|  | Binge eating behavior | 1.24 (-4.50, 6.98) | 2.84 (-1.75, 7.43) | 1.81 (-2.56, 6.18) | 0.97 (-2.70, 4.64) |
|  | Overeating behavior without LOC | 0.0 (ref) | 0.0 (ref) | 0.0 (ref) | 0.0 (ref) |
|  | No overeating behavior | **-5.07 (-9.09, -1.04)** | -2.95 (-6.19, 0.29) | **-3.19 (-6.25, -0.13)** | -2.50 (-5.07, 0.07) |
| Systolic BP, mmHg | |  |  |  |  |
|  | Binge eating behavior | 3.86 (-1.35, 9.08) | 2.46 (-2.08, 7.00) | 2.35 (-2.18, 6.88) | 2.13 (-2.34, 6.61) |
|  | Overeating behavior without LOC | 0.0 (ref) | 0.0 (ref) | 0.0 (ref) | 0.0 (ref) |
|  | No overeating behavior | 3.34 (-0.14, 6.83) | 1.23 (-1.85, 4.31) | 1.21 (-1.86, 4.28) | 1.67 (-1.37, 4.71) |
| Diastolic BP, mmHg | |  |  |  |  |
|  | Binge eating behavior | 1.83 (-2.18, 5.83) | 2.72 (-1.27, 6.72) | 2.64 (-1.34, 6.63) | 2.47 (-1.48, 6.42) |
|  | Overeating behavior without LOC | 0.0 (ref) | 0.0 (ref) | 0.0 (ref) | 0.0 (ref) |
|  | No overeating behavior | -0.18 (-2.85, 2.50) | 0.61 (-2.10, 3.32) | 0.64 (-2.06, 3.35) | 1.00 (-1.68, 3.68) |
| *Log-transformed outcomes* |  |  | | | |
| ALT, U/L | |  |  |  |  |
|  | Binge eating behavior | 0.11 (-0.20, 0.43) | 0.08 (-0.22, 0.39) | 0.06 (-0.25, 0.38) | 0.08 (-0.23, 0.39) |
|  | Overeating behavior without LOC | 0.0 (ref) | 0.0 (ref) | 0.0 (ref) | 0.0 (ref) |
|  | No overeating behavior | -0.03 (-0.26, 0.21) | -0.07 (-0.30, 0.16) | -0.07 (-0.31, 0.16) | -0.05 (-0.28, 0.18) |
| Total cholesterol, mg/dL | |  |  |  |  |
|  | Binge eating behavior | 0.06 (-0.10, 0.22) | 0.08 (-0.07, 0.24) | 0.10 (-0.06, 0.26) | 0.10 (-0.05, 0.26) |
|  | Overeating behavior without LOC | 0.0 (ref) | 0.0 (ref) | 0.0 (ref) | 0.0 (ref) |
|  | No overeating behavior | -0.02 (-0.13, 0.10) | 0.01 (-0.11, 0.13) | 0.01 (-0.11, 0.13) | 0.02 (-0.10, 0.14) |
| HDL, mg/dL | |  |  |  |  |
|  | Binge eating behavior | -0.03 (-0.22, 0.16) | -0.01 (-0.19, 0.17) | 0.02 (-0.15, 0.20) | 0.01 (-0.16, 0.19) |
|  | Overeating behavior without LOC | 0.0 (ref) | 0.0 (ref) | 0.0 (ref) | 0.0 (ref) |
|  | No overeating behavior | -0.02 (-0.16, 0.12) | 0.02 (-0.12, 0.16) | 0.03 (-0.10, 0.16) | 0.01 (-0.12, 0.14) |
| Triglycerides, mg/dL | |  |  |  |  |
|  | Binge eating behavior | 0.09 (-0.24, 0.41) | 0.09 (-0.23, 0.41) | 0.08 (-0.24, 0.41) | 0.09 (-0.23, 0.42) |
|  | Overeating behavior without LOC | 0.0 (ref) | 0.0 (ref) | 0.0 (ref) | 0.0 (ref) |
|  | No overeating behavior | 0.02 (-0.21, 0.26) | 0.00 (-0.24, 0.24) | -0.01 (-0.24, 0.23) | 0.01 (-0.23, 0.24) |
| IL-6, pg/mL | |  |  |  |  |
|  | Binge eating behavior | 0.20 (-0.36, 0.76) | 0.27 (-0.30, 0.83) | 0.21 (-0.36, 0.78) | 0.22 (-0.34, 0.79) |
|  | Overeating behavior without LOC | 0.0 (ref) | 0.0 (ref) | 0.0 (ref) | 0.0 (ref) |
|  | No overeating behavior | **-0.43 (-0.85, -0.02)** | -0.33 (-0.75, 0.09) | -0.33 (-0.76, 0.09) | -0.30 (-0.73, 0.12) |
| hsCRP, mg/L | |  |  |  |  |
|  | Binge eating behavior | -0.55 (-1.78, 0.68) | -0.44 (-1.68, 0.80) | -0.76 (-1.98, 0.46) | -0.68 (-1.87, 0.51) |
|  | Overeating behavior without LOC | 0.0 (ref) | 0.0 (ref) | 0.0 (ref) | 0.0 (ref) |
|  | No overeating behavior | **-1.24 (-2.14, -0.33)** | **-1.02 (-1.96, -0.08)** | **-1.06 (-1.98, -0.15)** | **-0.93 (-1.83, -0.04)** |
| Insulin, uU/ml | |  |  |  |  |
|  | Binge eating behavior | 0.27 (-0.20, 0.73) | 0.33 (-0.13, 0.79) | 0.24 (-0.22, 0.71) | 0.30 (-0.15, 0.74) |
|  | Overeating behavior without LOC | 0.0 (ref) | 0.0 (ref) | 0.0 (ref) | 0.0 (ref) |
|  | No overeating behavior | -0.09 (-0.42, 0.24) | 0.02 (-0.33, 0.36) | 0.01 (-0.33, 0.35) | 0.06 (-0.27, 0.39) |
| Glucose, mg/dL | |  |  |  |  |
|  | Binge eating behavior | 0.03 (-0.02, 0.08) | 0.03 (-0.02, 0.08) | 0.03 (-0.02, 0.08) | 0.03 (-0.02, 0.08) |
|  | Overeating behavior without LOC | 0.0 (ref) | 0.0 (ref) | 0.0 (ref) | 0.0 (ref) |
|  | No overeating behavior | 0.00 (-0.04, 0.03) | 0.00 (-0.04, 0.03) | 0.00 (-0.04, 0.03) | 0.00 (-0.04, 0.03) |
| HOMA-IR, units | |  |  |  |  |
|  | Binge eating behavior | 0.36 (-0.12, 0.84) | 0.42 (-0.07, 0.90) | 0.31 (-0.17, 0.79) | 0.35 (-0.12, 0.81) |
|  | Overeating behavior without LOC | 0.0 (ref) | 0.0 (ref) | 0.0 (ref) | 0.0 (ref) |
|  | No overeating behavior | -0.13 (-0.47, 0.21) | -0.02 (-0.37, 0.33) | -0.04 (-0.38, 0.31) | 0.03 (-0.31, 0.36) |
| HbA1c, percent | |  |  |  |  |
|  | Binge eating behavior | 0.01 (-0.04, 0.06) | 0.02 (-0.03, 0.07) | 0.02 (-0.03, 0.07) | 0.02 (-0.03, 0.07) |
|  | Overeating behavior without LOC | 0.0 (ref) | 0.0 (ref) | 0.0 (ref) | 0.0 (ref) |
|  | No overeating behavior | -0.02 (-0.06, 0.02) | -0.01 (-0.05, 0.03) | -0.01 (-0.05, 0.03) | -0.01 (-0.05, 0.03) |
| Leptin, ng/mL | |  |  |  |  |
|  | Binge eating behavior | 0.39 (-0.68, 1.45) | 0.62 (-0.19, 1.43) | 0.31 (-0.49, 1.11) | 0.43 (-0.25, 1.11) |
|  | Overeating behavior without LOC | 0.0 (ref) | 0.0 (ref) | 0.0 (ref) | 0.0 (ref) |
|  | No overeating behavior | -0.39 (-1.19, 0.41) | 0.03 (-0.60, 0.65) | -0.11 (-0.71, 0.50) | 0.09 (-0.43, 0.61) |
| Adiponectin, ug/mL | |  |  |  |  |
|  | Binge eating behavior | -0.29 (-0.68, 0.10) | -0.28 (-0.64, 0.09) | -0.25 (-0.62, 0.12) | -0.26 (-0.63, 0.10) |
|  | Overeating behavior without LOC | 0.0 (ref) | 0.0 (ref) | 0.0 (ref) | 0.0 (ref) |
|  | No overeating behavior | 0.15 (-0.14, 0.43) | 0.16 (-0.12, 0.43) | 0.17 (-0.11, 0.44) | 0.14 (-0.14, 0.41) |

Model 1. Unadjusted

Model 2. Model 1 + adjustment for participant factors (sex, race/ethnicity, age at late adolescent visit)

Model 3. Model 2 + maternal factors (education, pre-pregnancy BMI, smoked during pregnancy, and excessive GWG)

Model 4. Model 3 + participant BMI z-score at early adolescent visit

**^†^**Binge eating behavior defined as a “yes” answer to: “In the past 12 months, have you ever eaten so much food in a short period of time that you would be embarrassed if others saw you?” AND “yes” to: “During the times when you ate this way, did you feel you couldn't stop eating or control what or how much you were eating?”

**^††^**Overeating behavior without LOC defined as “no” response to the second question.

Bold text identifies those values for which the 95% CI does not include 0.0.

LOC = loss of control, BMI = body mass index, BIA = bioelectrical impedance analysis, DXA = dual-energy X-ray absorptiometry, BP = blood pressure, ALT = alanine aminotransferase, HDL = high-density lipoprotein, IL-6 = interleukin-6, hsCRP = high-sensitivity C-reactive protein, HOMA-IR = homeostatic model assessment of insulin resistance, HbA1c = hemoglobin A1c, GWG = gestational weight gain.

**Supplemental Table 4.** Multivariable regression models assessing the associations of overeating behavior† in early adolescence, with cardiometabolic outcomes in late adolescence among adolescents in the Project Viva cohort, stratified by sex at birth

|  | **Females** | | **Males** | |  | |  |
| --- | --- | --- | --- | --- | --- | --- | --- |
| ***Late adolescent outcomes*** | **Model 3** | **Model 4** | **Model 3** | **Model 4** | **Model 3** | **Model 4** | |
|  | ß (95% CI) | | | | P values for interaction | | |
| BMI z-score, units | **0.37 (0.06, 0.68)** | 0.10 (-0.11, 0.31) | 0.15 (-0.34, 0.64) | -0.03 (-0.35, 0.30) | 0.30 | 0.71 | |
| Waist circumference, cm | 3.99 (-0.04, 8.02) | 1.23 (-2.10, 4.55) | 1.73 (-3.99, 7.44) | 0.02 (-4.42, 4.47) | 0.48 | 0.93 | |
| Percent fat by BIA | **3.38 (0.64, 6.12)** | 1.31 (-0.83, 3.46) | 0.92 (-2.66, 4.50) | -0.14 (-2.95, 2.67) | 0.24 | 0.51 | |
| Percent fat by DXA | **4.40 (1.73, 7.06)** | **3.04 (0.79, 5.29)** | 3.53 (-0.64, 7.70) | 3.38 (-0.13, 6.89) | 0.74 | 0.67 | |
| Systolic BP, mmHg | -0.62 (-3.49, 2.26) | -0.85 (-3.74, 2.05) | -0.48 (-4.68, 3.73) | -0.95 (-5.05, 3.15) | 0.94 | 0.92 | |
| Diastolic BP, mmHg | -0.01 (-2.51, 2.49) | -0.33 (-2.84, 2.18) | 0.99 (-2.75, 4.74) | 0.67 (-3.02, 4.37) | 0.71 | 0.59 | |
| ***Log-transformed outcomes*** |  |  |  |  |  |  | |
| ALT, U/L | 0.13 (-0.06, 0.32) | 0.12 (-0.07, 0.31) | 0.05 (-0.26, 0.36) | 0.06 (-0.25, 0.36) | 0.97 | 0.87 | |
| Total cholesterol, mg/dL | 0.06 (-0.04, 0.15) | 0.06 (-0.04, 0.15) | 0.01 (-0.15, 0.16) | 0.01 (-0.15, 0.16) | 0.67 | 0.72 | |
| HDL, mg/dL | 0.02 (-0.10, 0.15) | 0.04 (-0.08, 0.16) | -0.09 (-0.25, 0.07) | -0.09 (-0.25, 0.06) | 0.30 | 0.20 | |
| Triglycerides, mg/dL | 0.06 (-0.16, 0.28) | 0.05 (-0.17, 0.27) | 0.12 (-0.18, 0.42) | 0.12 (-0.18, 0.42) | 0.58 | 0.51 | |
| IL-6, pg/mL | 0.25 (-0.11, 0.61) | 0.23 (-0.13, 0.59) | 0.64 (0.10, 1.17) | 0.64 (0.11, 1.17) | 0.25 | 0.20 | |
| hsCRP, mg/L | 0.45 (-0.39, 1.29) | 0.33 (-0.48, 1.15) | 0.74 (-0.37, 1.84) | 0.75 (-0.33, 1.84) | 0.85 | 0.64 | |
| Insulin, uU/ml | 0.14 (-0.18, 0.46) | 0.12 (-0.20, 0.45) | 0.04 (-0.37, 0.45) | 0.05 (-0.33, 0.43) | 0.72 | 0.90 | |
| Glucose, mg/dL | 0.01 (-0.02, 0.05) | 0.02 (-0.02, 0.05) | 0.03 (-0.02, 0.07) | 0.03 (-0.02, 0.07) | 0.54 | 0.52 | |
| HOMA-IR, units | 0.25 (-0.08, 0.58) | 0.22 (-0.11, 0.56) | 0.10 (-0.34, 0.53) | 0.10 (-0.30, 0.50) | 0.71 | 0.94 | |
| HbA1c, percent | 0.01 (-0.03, 0.04) | 0.01 (-0.03, 0.04) | 0.05 (0.00, 0.09) | 0.05 (0.00, 0.09) | 0.30 | 0.33 | |
| Leptin, ng/mL | 0.35 (-0.08, 0.79) | 0.24 (-0.14, 0.62) | 0.17 (-0.66, 1.00) | 0.20 (-0.48, 0.88) | 0.78 | 0.72 | |
| Adiponectin, ug/mL | **-0.30 (-0.56, -0.04)** | **-0.27 (-0.53, -0.01)** | -0.24 (-0.57, 0.09) | -0.24 (-0.57, 0.09) | 0.84 | 0.98 | |

Model 3. Adjustment for participant demographic factors (sex, race/ethnicity, age at late adolescent visit) + maternal factors (education, pre-pregnancy BMI, smoked during pregnancy, and excessive GWG)

Model 4. Model 3 + participant BMI z-score at early adolescent visit

†Overeating behavior defined as a “yes” answer to: “In the past 12 months, have you ever eaten so much food in a short period of time that you would be embarrassed if others saw you?”

Bold text identifies those values for which the 95% CI does not include 0.0.

BMI = body mass index, BIA = bioelectrical impedance analysis, DXA = dual-energy X-ray absorptiometry, BP = blood pressure, IQR = interquartile range, ALT = alanine aminotransferase, HDL = high-density lipoprotein, IL-6 = interleukin-6, hsCRP = high-sensitivity C-reactive protein, HOMA-IR = homeostatic model assessment of insulin resistance, HbA1c = hemoglobin A1c, GWG = gestational weight gain.

**Supplemental Table 5.** Multivariable regression models assessing the associations of overeating behavior† in early adolescence, with cardiometabolic outcomes in late adolescence among adolescents in the Project Viva cohort, stratified by body mass index category in early adolescence.

|  | **BMI <85th %tile in early adolescence** | | **BMI ≥85th %tile in early adolescence** | |  |  |
| --- | --- | --- | --- | --- | --- | --- |
| ***Late adolescent outcomes*** | **Model 3** | **Model 4** | **Model 3** | **Model 4** | **Model 3** | **Model 4** |
|  | ß (95% CI) | | | | P values for interaction | |
| BMI z-score, units | **0.38 (0.08, 0.68)** | 0.19 (-0.04, 0.42) | -0.17 (-0.52, 0.18) | -0.21 (-0.51, 0.08) | **0.04** | 0.11 |
| Waist circumference, cm | **3.00 (0.38, 5.63)** | 2.02 (-0.41, 4.44) | -2.02 (-8.47, 4.43) | -2.98 (-8.06, 2.10) | 0.22 | 0.40 |
| Percent fat by BIA | **3.15 (1.02, 5.27)** | **2.18 (0.29, 4.07)** | -1.67 (-5.33, 2.00) | -2.16 (-5.21, 0.89) | **0.04** | 0.10 |
| Percent fat by DXA | **3.24 (1.06, 5.43)** | **2.89 (0.80, 4.97)** | 2.37 (-1.87, 6.62) | 1.86 (-1.98, 5.71) | 0.82 | 0.98 |
| Systolic BP, mmHg | -0.96 (-4.00, 2.08) | -1.48 (-4.48, 1.51) | 0.40 (-3.65, 4.44) | 0.42 (-3.64, 4.48) | 0.72 | 0.59 |
| Diastolic BP, mmHg | 0.11 (-2.53, 2.75) | -0.03 (-2.67, 2.61) | -0.10 (-3.71, 3.51) | -0.26 (-3.82, 3.30) | 0.95 | 0.98 |
| ***Log-transformed outcomes*** |  |  |  |  |  |  |
| ALT, U/L | 0.15 (-0.06, 0.36) | 0.15 (-0.06, 0.36) | -0.07 (-0.37, 0.23) | -0.07 (-0.37, 0.23) | 0.64 | 0.59 |
| Total cholesterol, mg/dL | 0.04 (-0.08, 0.15) | 0.04 (-0.08, 0.15) | -0.03 (-0.16, 0.10) | -0.03 (-0.17, 0.10) | 0.94 | 0.93 |
| HDL, mg/dL | -0.03 (-0.15, 0.10) | -0.03 (-0.15, 0.10) | 0.01 (-0.13, 0.15) | 0.03 (-0.12, 0.17) | 0.43 | 0.41 |
| Triglycerides, mg/dL | 0.07 (-0.17, 0.30) | 0.07 (-0.17, 0.30) | 0.02 (-0.28, 0.31) | 0.00 (-0.30, 0.31) | 0.57 | 0.58 |
| IL-6, pg/mL | 0.37 (-0.05, 0.79) | 0.37 (-0.06, 0.79) | 0.36 (-0.10, 0.82) | 0.32 (-0.15, 0.79) | 0.81 | 0.81 |
| hsCRP, mg/L | 0.73 (-0.10, 1.56) | 0.74 (-0.10, 1.57) | 0.30 (-0.85, 1.46) | 0.10 (-1.01, 1.21) | 0.56 | 0.51 |
| Insulin, uU/ml | 0.15 (-0.18, 0.49) | 0.16 (-0.18, 0.49) | 0.12 (-0.24, 0.47) | 0.10 (-0.26, 0.45) | 0.87 | 0.83 |
| Glucose, mg/dL | 0.02 (-0.01, 0.06) | 0.02 (-0.01, 0.06) | 0.00 (-0.04, 0.04) | 0.00 (-0.05, 0.04) | 0.50 | 0.49 |
| HOMA-IR, units | 0.25 (-0.10, 0.60) | 0.25 (-0.10, 0.60) | 0.12 (-0.26, 0.51) | 0.10 (-0.29, 0.48) | 0.64 | 0.61 |
| HbA1c, percent | 0.01 (-0.02, 0.05) | 0.01 (-0.02, 0.05) | 0.02 (-0.03, 0.07) | 0.02 (-0.03, 0.07) | 0.43 | 0.39 |
| Leptin, ng/mL | 0.21 (-0.31, 0.72) | 0.22 (-0.27, 0.71) | 0.07 (-0.55, 0.69) | -0.07 (-0.61, 0.47) | 0.74 | 0.59 |
| Adiponectin, ug/mL | **-0.37 (-0.62, -0.11)** | **-0.37 (-0.62, -0.11)** | -0.14 (-0.50, 0.22) | -0.11 (-0.47, 0.24) | 0.37 | 0.35 |

Model 3. Adjustment for participant demographic factors (sex, race/ethnicity, age at late adolescent visit) + maternal factors (education, pre-pregnancy BMI, smoked during pregnancy, and excessive GWG)

Model 4. Model 3 + participant BMI z-score at early adolescent visit

†Overeating behavior defined as a “yes” answer to: “In the past 12 months, have you ever eaten so much food in a short period of time that you would be embarrassed if others saw you?”

Bold text identifies those values for which the 95% CI does not include 0.0.

BMI = body mass index, BIA = bioelectrical impedance analysis, DXA = dual-energy X-ray absorptiometry, BP = blood pressure, IQR = interquartile range, ALT = alanine aminotransferase, HDL = high-density lipoprotein, IL-6 = interleukin-6, hsCRP = high-sensitivity C-reactive protein, HOMA-IR = homeostatic model assessment of insulin resistance, HbA1c = hemoglobin A1c, GWG = gestational weight gain.
